# The Lipid Raft-Associated Protein Stomatin Is Required for Accumulation of Dectin-1 in the Phagosomal Membrane and for Full Activity of Macrophages against Aspergillus fumigatus

**DOI:** 10.1128/msphere.00523-22

**Published:** 2023-01-31

**Authors:** Marie Goldmann, Franziska Schmidt, Zoltán Cseresnyés, Thomas Orasch, Susanne Jahreis, Susann Hartung, Marc Thilo Figge, Marie von Lilienfeld-Toal, Thorsten Heinekamp, Axel A. Brakhage

**Affiliations:** a Department of Molecular and Applied Microbiology, Leibniz Institute for Natural Product Research and Infection Biology—Hans Knöll Institute (Leibniz-HKI), Jena, Germany; b Department of Microbiology and Molecular Biology, Institute of Microbiology, Friedrich Schiller University Jena, Jena, Germany; c Research Group Applied Systems Biology, Leibniz-HKI, Jena, Germany; d Department of Infections in Hematology/Oncology, Leibniz-HKI, Jena, Germany; e Institute of Microbiology, Faculty of Biological Sciences, Friedrich Schiller University Jena, Jena, Germany; f Department of Hematology and Medical Oncology, Jena University Hospital, Jena, Germany; University of Georgia

**Keywords:** *Aspergillus fumigatus*, CRISPR/Cas9, dectin-1, phagosomal maturation, lipid rafts, phagocytosis, phagosomes, stomatin

## Abstract

Alveolar macrophages belong to the first line of defense against inhaled conidia of the human-pathogenic fungus Aspergillus fumigatus. In lung alveoli, they contribute to phagocytosis and elimination of conidia. As a counterdefense, conidia have a gray-green pigment that enables them to survive in phagosomes of macrophages for some time. Previously, we showed that this conidial pigment interferes with the formation of flotillin-dependent lipid raft microdomains in the phagosomal membrane, thereby preventing the formation of functional phagolysosomes. Besides flotillins, stomatin is a major component of lipid rafts and can be targeted to the membrane. However, only limited information on stomatin is available, in particular on its role in defense against pathogens. To determine the function of this integral membrane protein, a stomatin-deficient macrophage line was generated by CRISPR/Cas9 gene editing. Immunofluorescence microscopy and flow cytometry revealed that stomatin contributes to the phagocytosis of conidia and is important for recruitment of the β-glucan receptor dectin-1 to both the cytoplasmic membrane and phagosomal membrane. In stomatin knockout cells, fusion of phagosomes and lysosomes, recruitment of the vATPase to phagosomes, and tumor necrosis factor alpha (TNF-α) levels were reduced when cells were infected with pigmentless conidia. Thus, our data suggest that stomatin is involved in maturation of phagosomes via fostering fusion of phagosomes with lysosomes.

**IMPORTANCE** Stomatin is an integral membrane protein that contributes to the uptake of microbes, e.g., spores of the human-pathogenic fungus Aspergillus fumigatus. By generation of a stomatin-deficient macrophage line by advanced genetic engineering, we found that stomatin is involved in the recruitment of the β-glucan receptor dectin-1 to the phagosomal membrane of macrophages. Furthermore, stomatin is involved in maturation of phagosomes via fostering fusion of phagosomes with lysosomes. The data provide new insights on the important role of stomatin in the immune response against human-pathogenic fungi.

## INTRODUCTION

Due to advances in modern medicine, in recent decades the number of immunocompromised patients has increased. In this patient cohort, a frequently diagnosed fungal infection is caused by the ubiquitous saprophytic mold Aspergillus fumigatus. The fungus produces myriad conidia, which are released into the atmosphere. With a diameter of 2 to 3 μm, conidia are inhaled by humans and can easily reach the lung alveoli (1, 2). Although conidia are apparently effectively cleared by the innate immune system, in patients with impaired immunity, conidia can escape the residual host defense. Outgrowing hyphae can then invade pulmonary tissue to cause invasive aspergillosis (3). Therefore, the characterization of the pathogen’s defense strategies against human immune responses and the identification of relevant host factors against A. fumigatus are of considerable interest.

In lung alveoli, resident alveolar macrophages belong to the first line of defense against inhaled conidia. The central role of macrophages is the internalization and degradation of microbial pathogens (4, 5). Reduced numbers or impaired activity of phagocytes, e.g., due to leukemia or after stem cell transplantation, increase susceptibility of patients to invasive aspergillosis (6–8).

Macrophages recognize conidia via pathogen recognition receptors (PRRs) and engulf them by forming a phagocytic cup. Within the macrophage, the conidium-containing phagosome fuses with lysosomes to form a phagolysosome (8, 9). This maturation process leads to an acidic pH inside the phagolysosome, which activates hydrolytic enzymes to eliminate the pathogen (10, 11). The conidial surface pigment dihydroxynaphthalene (DHN)-melanin plays a crucial role in host pathogen interaction. It interferes with phagocytic uptake and inhibits phagosomal acidification (12, 13). In line, conidia lacking the polyketide synthase PksP, catalyzing the initial step of DHN-melanin biosynthesis (14), are less resistant against the immune response. Melanin-free conidia show higher phagocytosis rates, an increased number of phagosome-lysosome fusion events, and enhanced acidification of phagolysosomes. This is associated with attenuated virulence of *pksP* mutant conidia in murine infection models (12, 14–16). We previously identified the mechanism of how DHN melanin interferes with the phagosome maturation: melanin inhibits the formation of flotillin-dependent lipid raft microdomains in the phagosomal membrane, which are required for formation of fully functional phagolysosomes, including the assembly of protein complexes like NADPH oxidase and vATPase (17).

Lipid rafts are small, heterogeneous, and highly dynamic structures within cell membranes (18). These sphingolipid- and cholesterol-rich microdomains preferentially contain glycosylphosphatidylinositol (GPI)-anchored proteins and integral proteins like flotillin-1, flotillin-2, and stomatin (19). In general, lipid rafts play important roles in response to intracellular or extracellular stimuli (20, 21). However, the precise roles of integral proteins for formation and dynamics of lipid raft microdomains await further analyses. In this regard, only limited information on stomatin is available, in particular on its role in defense against pathogens.

Stomatin is conserved from archaea to mammals (22). Consistent with other proteins involved in membrane organization, stomatin proteins form higher-order oligomers and are enriched in lipid raft microdomains (23, 24). Stomatin-like and related proteins are characterized by an SPFH (stomatin, prohibitin, flotillin, and HfIK/C) domain, which is crucial for its association with the membrane scaffold, the cellular cytoskeleton, and other membrane proteins (25–27). Stomatin is a major component of lipid rafts and can be targeted to the membrane (19, 23, 28–30). To shed light on this central protein, we characterized in detail the basic functions of stomatin in macrophages, with an emphasis on its role in recognition of and defense against A. fumigatus.

## RESULTS

### Phagosomal membranes of RAW264.7 macrophages and BMDMs contain stomatin.

**FIG 1:**
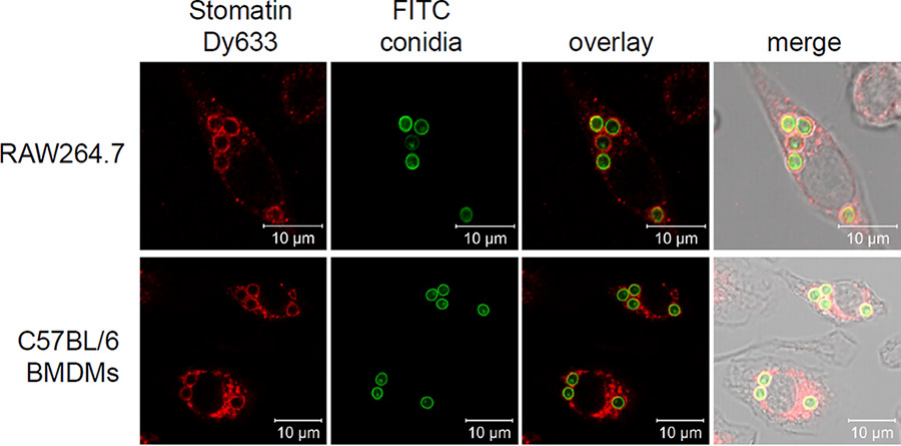
Localization of stomatin at phagosomal membranes of RAW264.7 macrophages and bone marrow-derived macrophages (BMDMs) of C57BL/6 mice after infection with A. fumigatus wild-type conidia (FITC labeled). Stomatin was detected by immunofluorescence using a stomatin antibody coupled to Dy633.

In general, lipid rafts are cholesterol- and sphingolipid-rich microdomains containing not only GPI-anchored proteins but also integral membrane proteins like flotillins and stomatin (19). Since the function of lipid raft microdomains for phagosome biogenesis is not yet fully understood, in this study, we characterized the potential role of stomatin in this process. Stomatin is ubiquitously expressed in human tissue (22). Targeting to the membrane is mediated by an N-terminal membrane insertion domain (31). Together with the stomatin-like protein 2 (SLP-2), stomatin forms hetero-oligomers at endosomal membranes (32). In line with this, by immunofluorescence we detected stomatin in the phagosomal membrane of RAW264.7 macrophages after infection with A. fumigatus conidia (Fig. 1). The same observation was made with primary mouse macrophages (C57BL/6 bone marrow-derived macrophages [BMDMs]) (Fig. 1). These data indicate that conidia reside in phagosomes containing stomatin in their membrane.

### CRISPR-Cas9 gene editing in RAW264.7 cells for generation of knockout cell lines.

**FIG 2:**
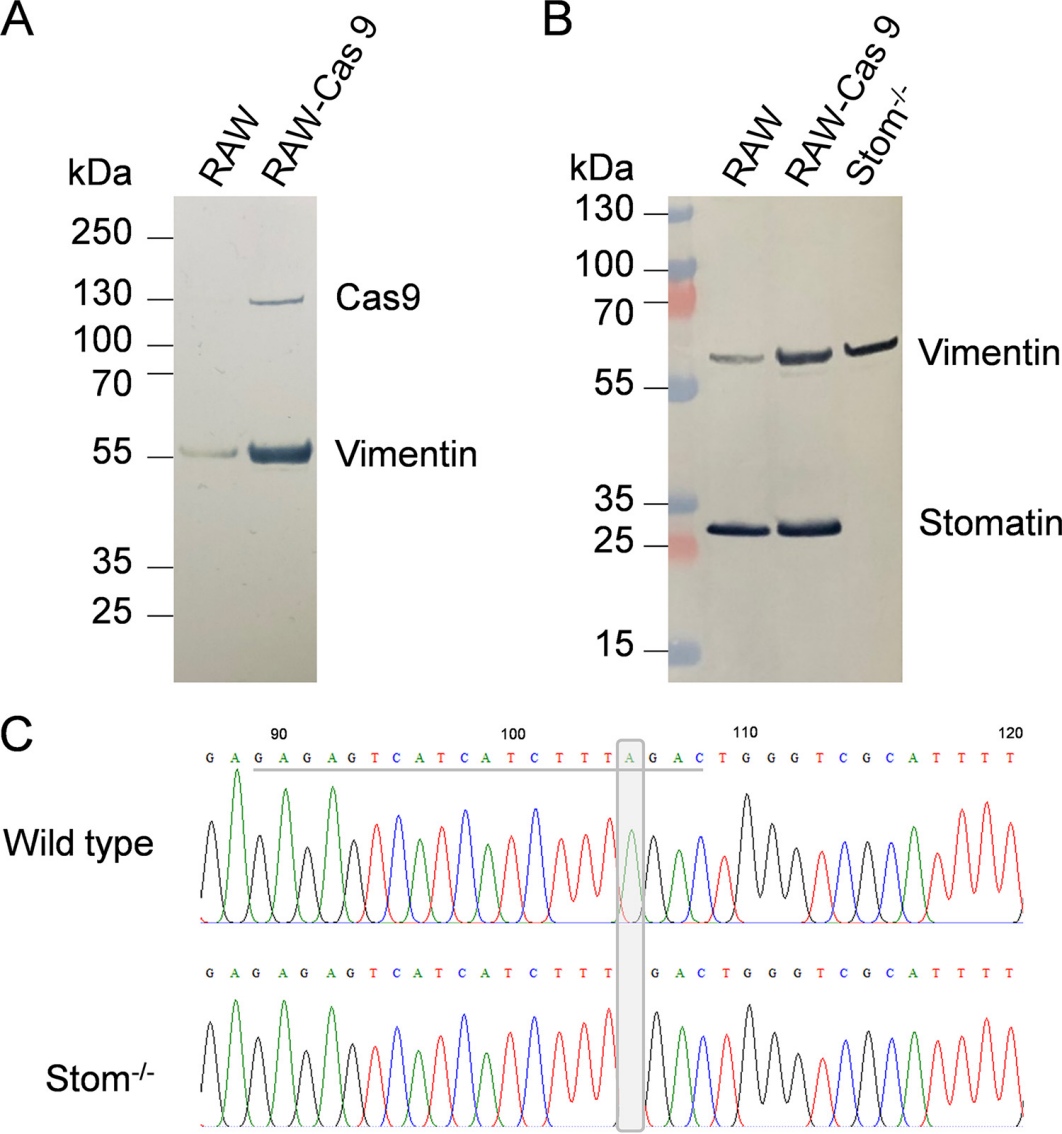
Generation of Stom^−/−^ cells. (A) Identification of a RAW264.7 macrophage line with stable expression of Cas9. RAW264.7 cells were transduced with Cas9-lentivirus. A selected puromycin-resistant clone was analyzed by Western blotting for production of Cas9. Vimentin was used as a control. (B) Western blot analysis to verify loss of stomatin protein in Stom^−/−^ RAW264.7 macrophages. (C) Sequence analysis of Stom^−/−^ macrophages. CRISPR/Cas9 gene editing resulted in loss of an adenine (gray box) compared to the wild-type sequence. The sgRNA target sequence is underlined.

For functional analysis of stomatin, a knockout cell line for stomatin was created by CRISPR-Cas9 gene editing. First, a stable macrophage line producing Cas9 was generated by transduction of RAW264.7 cells with Cas9 lentivirus particles. The expression of the Cas9 protein was confirmed by immunoblotting (Fig. 2A). These cells were further transduced with single guide RNA (sgRNA) lentivirus particles targeting exon 3 of the *Stom* gene. Hygromycin-resistant clones were screened and the knockout of stomatin in selected clones was verified by Western blotting (Fig. 2B). Sequence analysis of a selected clone that was used for all further studies revealed deletion of an adenine at position 199 of the coding *Stom* sequence, resulting in a frameshift and consequently in stomatin-deficient cells (Fig. 2C).

### Reduced phagocytosis of A. fumigatus *pksP* conidia by Stom^−/−^ macrophages.

**FIG 3:**
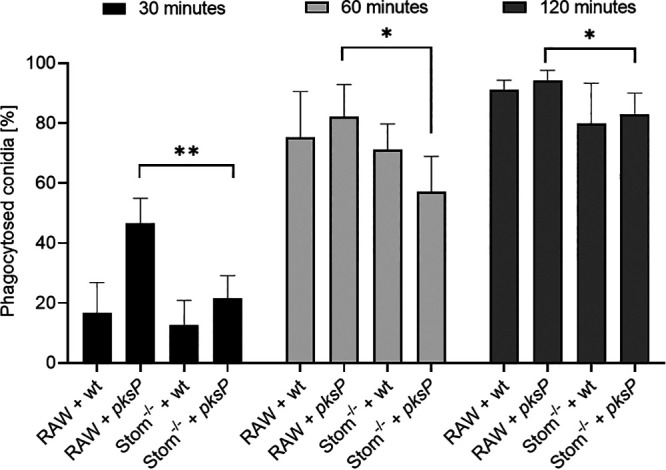
Percentage of phagocytosed A. fumigatus wild-type (wt) and *pksP* conidia after coincubation for 30, 60, and 120 min with RAW264.7 or Stom^−/−^ macrophages.

Phagocytosis of conidia depends on recognition of A. fumigatus by pattern recognition receptors (PRRs) and the reorganization of the membrane structure to form a phagocytic cup and to engulf the pathogen (33–35). To analyze whether the knockout of stomatin affects this process, the kinetics of phagocytosis of fluorescein isothiocyanate (FITC)-labeled A. fumigatus conidia was monitored over a period of 30 to 120 min (Fig. 3). After 30 min, in the Stom^−/−^ macrophages the percentage of phagocytosed *pksP* conidia was reduced by 50% compared to that in RAW264.7 cells. Longer coincubation times, 60 and 120 min, led to an increase in the percentage of phagocytosed conidia. However, with regard to phagocytosis of *pksP* conidia, a significant difference between Stom^−/−^ and wild-type macrophages remained. No significant difference in the percentage of phagocytosed wild-type conidia was observed between stomatin knockout and wild-type cells.

**FIG 4:**
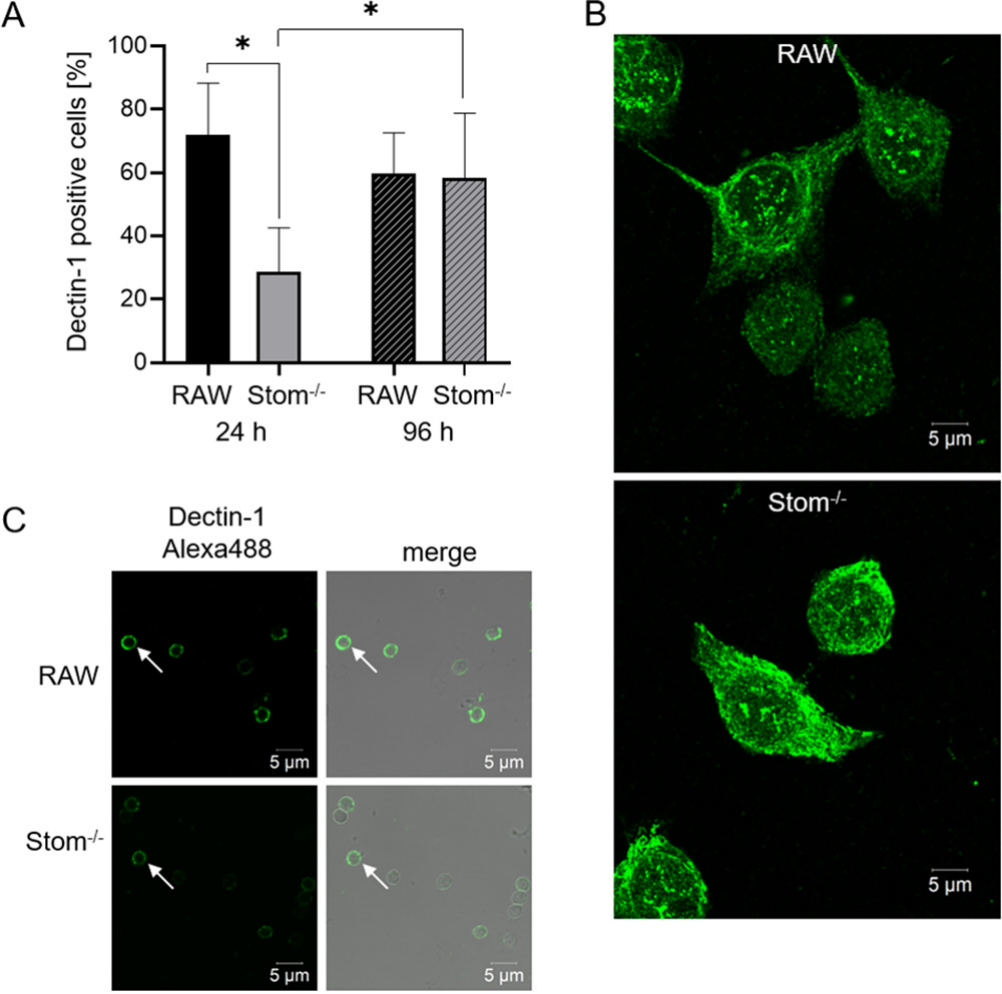
The role of interaction of dectin-1 and stomatin in host defense against A. fumigatus conidia. (A) Percentage of dectin-1-positive cells measured by flow cytometry after culture for 24 and 96 h. A comparison of RAW264.7 wild-type and Stom^−/−^ macrophages is shown. (B) Immunofluorescence of methanol-acetone-fixed RAW264.7 wild-type and Stom^−/−^ cells for dectin-1 (Alexa Fluor 488). High-resolution microscopic images were taken with Airyscan technology. (C) Immunofluorescence staining of dectin-1 (Alexa Fluor 488) on isolated phagolysosomes of wild-type and Stom^−/−^ macrophages after infection with A. fumigatus *pksP* conidia.

Because of the reduced numbers of phagocytosed *pksP* conidia in Stom^−/−^ cells after 30 and 60 min, we wondered whether stomatin influences the positioning of relevant PRRs in the membrane. Previously, it was shown that phagocytosis of conidia and in particular of *pksP* conidia depends on the receptor dectin-1, a C-type lectin. The ligand of dectin-1, β-1,3-glucan, is in particular readily accessible on *pksP* mutant conidia and also on swollen conidia (34, 36). Since Stom^−/−^ cells also phagocytose swollen conidia less efficiently than RAW264.7 cells (see Fig. S5 in the supplemental material), it was reasonable to assume that stomatin is required for the recruitment of dectin-1. We thus analyzed dectin-1 on membranes of macrophages by immunostaining and flow cytometry. After 24 h of cultivation, only 30% of Stom^−/−^ cells were found to be dectin-1 positive, compared to 70% of wild-type cells (Fig. 4A). However, after 96 h of cultivation, there was no difference anymore in the number of dectin-1-positive cells. To verify the observation of a different dectin-1 distribution in Stom^−/−^ cells than in RAW264.7 macrophages, the localization of dectin-1 was analyzed by immunofluorescence and high-resolution imaging. Wild-type macrophages showed a homogeneous, fine signal at the cell surface, whereas the signal in Stom^−/−^ cells appeared more compact and was localized in the cytoplasm rather than in the cytoplasmic membrane (Fig. 4B). These data suggest that stomatin is required for the timely and correct localization of dectin-1 on the cell surface.

10.1128/msphere.00523-22.5 FIG S5 Percentage of phagocytosed swollen A. fumigatus wild-type (wt) conidia after coincubation with RAW264.7 or Stom^−/−^ macrophages for 30 min compared to resting wild-type and *pksP* conidia. Download FIG S5, PDF file, 0.1 MB. Copyright © 2023 Goldmann et al. 2023 Goldmann et al. https://creativecommons.org/licenses/by/4.0/ This content is distributed under the terms of the Creative Commons Attribution 4.0 International license.

Besides recognition of fungal surface polysaccharides, dectin-1 is involved in phagosomal maturation processes (37). Therefore, the impact of stomatin on enrichment of dectin-1 on the phagosomal membrane surrounding conidia was determined by immunofluorescence. Isolated phagosomes of RAW264.7 macrophages with internalized *pksP* conidia showed a strong dectin-1 signal (Fig. 4C). In contrast, phagosomes of Stom^−/−^ macrophages revealed a weak dectin-1 signal after phagocytosis of *pksP* conidia, indicating that stomatin is required for quantitative accumulation of dectin-1 on the phagosomal membrane (Fig. 4C).

In a previous study, flotillins were identified as major chaperons for lipid raft microdomains during the intracellular processing of A. fumigatus conidia by phagocytes (17). To detect a possible regulatory link of dectin-1 and lipid rafts, the localization of flotillins in dectin-1-deficient cells was investigated. Compared to C57BL/6 wild-type macrophages, dectin-1-knockout cells showed reduced recruitment of flotillins to *pksP*-containing phagolysosomes (Fig. S6), suggesting a regulatory connection of dectin-1 and the formation of lipid raft microdomains.

10.1128/msphere.00523-22.6 FIG S6 Localization of flotillins in dectin-1-deficient cells. Fluorescence intensity of flotillin in conidia-containing phagolysosomes was measured in C57BL/6 wild-type macrophages and dectin-1 knockout cells. Download FIG S6, PDF file, 0.1 MB. Copyright © 2023 Goldmann et al. 2023 Goldmann et al. https://creativecommons.org/licenses/by/4.0/ This content is distributed under the terms of the Creative Commons Attribution 4.0 International license.

### Enrichment of sphingolipids in lipid raft microdomains is independent of stomatin.

**FIG 5:**
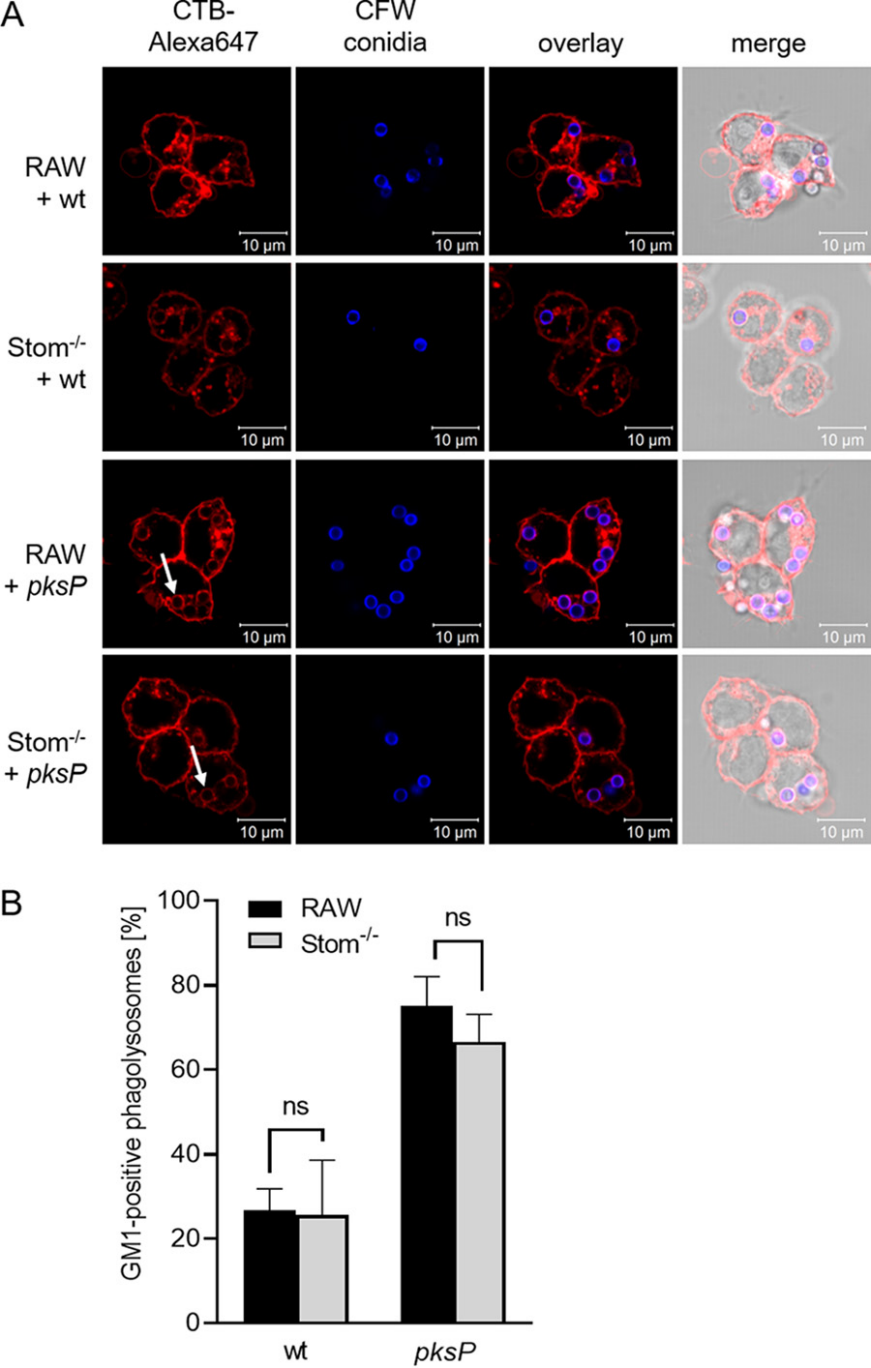
Enrichment of sphingolipids in lipid raft microdomains. (A) RAW264.7 and Stom^−/−^ macrophages were infected with CFW-labeled A. fumigatus wild-type or *pksP* conidia (CFW labeled; blue) and stained for GM1 ganglioside (CTB-Alexa Fluor 647; red); white arrows indicate phagosomes. (B) Percentage of GM1-positive phagosomes in RAW264.7 wild-type and Stom^−/−^ macrophages after infection with A. fumigatus wild-type or *pksP* conidia.

Lipid raft microdomains are enriched in cholesterol and sphingolipids. To examine whether the enrichment of sphingolipids in lipid raft microdomains is affected by stomatin, macrophages were infected with melanized wild-type and pigmentless *pksP* conidia and stained with Alexa Fluor 647-conjugated cholera toxin subunit B (CTB). CTB labels sphingolipid GM1 gangliosides, and accumulation of GM1 at distinct membrane sites is a characteristic feature of lipid rafts (38). RAW264.7 wild-type as well as Stom^−/−^ macrophages that had engulfed wild-type conidia revealed only faint CTB staining of the phagosomal membrane (Fig. 5A). A stronger CTB signal was detected for *pksP* conidia-containing phagosomes in wild-type macrophages than in Stom^−/−^ cells (Fig. 5A). In contrast, in Stom^−/−^ macrophages fainter CTB-mediated staining of the cytoplasmic membranes was detected. Quantification of the percentage of GM1-positive phagosomes revealed no significant differences between RAW264.7 wild-type and Stom^−/−^ macrophages (Fig. 5B).

### Stomatin deficiency impairs vATPase assembly and interferes with acidification of conidia-containing phagosomes.

**FIG 6:**
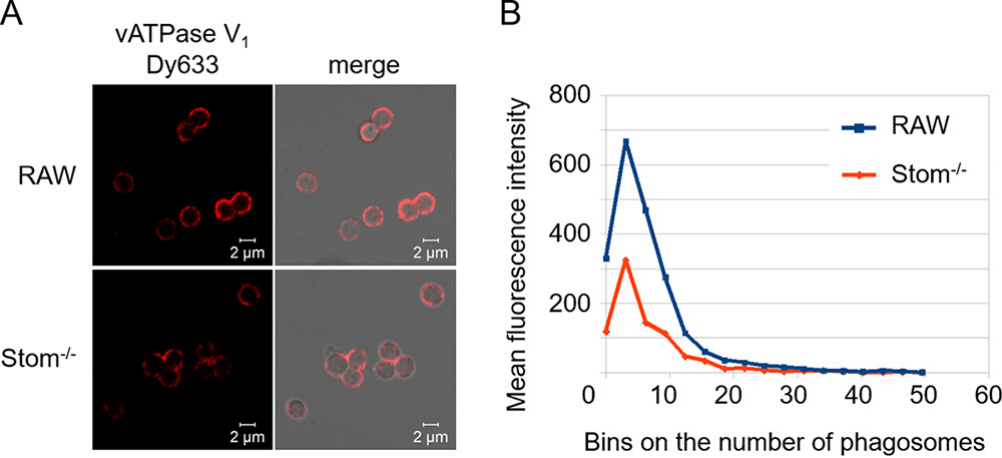
Impact of stomatin on vATPase assembly and acidification of phagosomes. (A) vATPase assembly in RAW264.7 and Stom^−/−^ macrophages after infection with A. fumigatus *pksP* conidia detected by immunofluorescence of V_1_ subunit. (B) Quantification of the mean fluorescence intensities for the vATPase of isolated phagosomes containing *pksP* mutant conidia.

It is known that in maturing phagosomes assembly of the vATPase complex takes place in membrane microdomains (39, 40). This process depends on flotillin-dependent lipid rafts (17). To determine whether stomatin is also involved in vATPase assembly, the percentage of the assembled protein complexes at the phagosomal membrane was quantified by immunofluorescence. As shown in Fig. 6A, the fluorescence due to the amount of subunit V_1_ assembled to the membranous V_0_ complex on the phagosomal membrane was affected by stomatin. Phagosomes of RAW264.7 macrophages containing *pksP* conidia display homogenous vATPase assembly at the phagosomal membrane (17). In contrast, the distribution of the signal at phagosomal membranes of Stom^−/−^ cells appeared more fragmented (Fig. 6A). Phagosomes of both wild-type and Stom^−/−^ cell lines that contained wild-type conidia showed only faint staining of the phagosomal membrane (data not shown). Image-based analyses of the signal intensities substantiated these findings. Phagosomes of Stom^−/−^ macrophages showed reduced fluorescence intensities after infection with *pksP* conidia compared to phagosomes from RAW264.7 wild-type cells (Fig. 6B), indicating a reduced number of assembled vATPase complexes in the phagosomal membrane of Stom^−/−^ macrophages.

**FIG 7:**
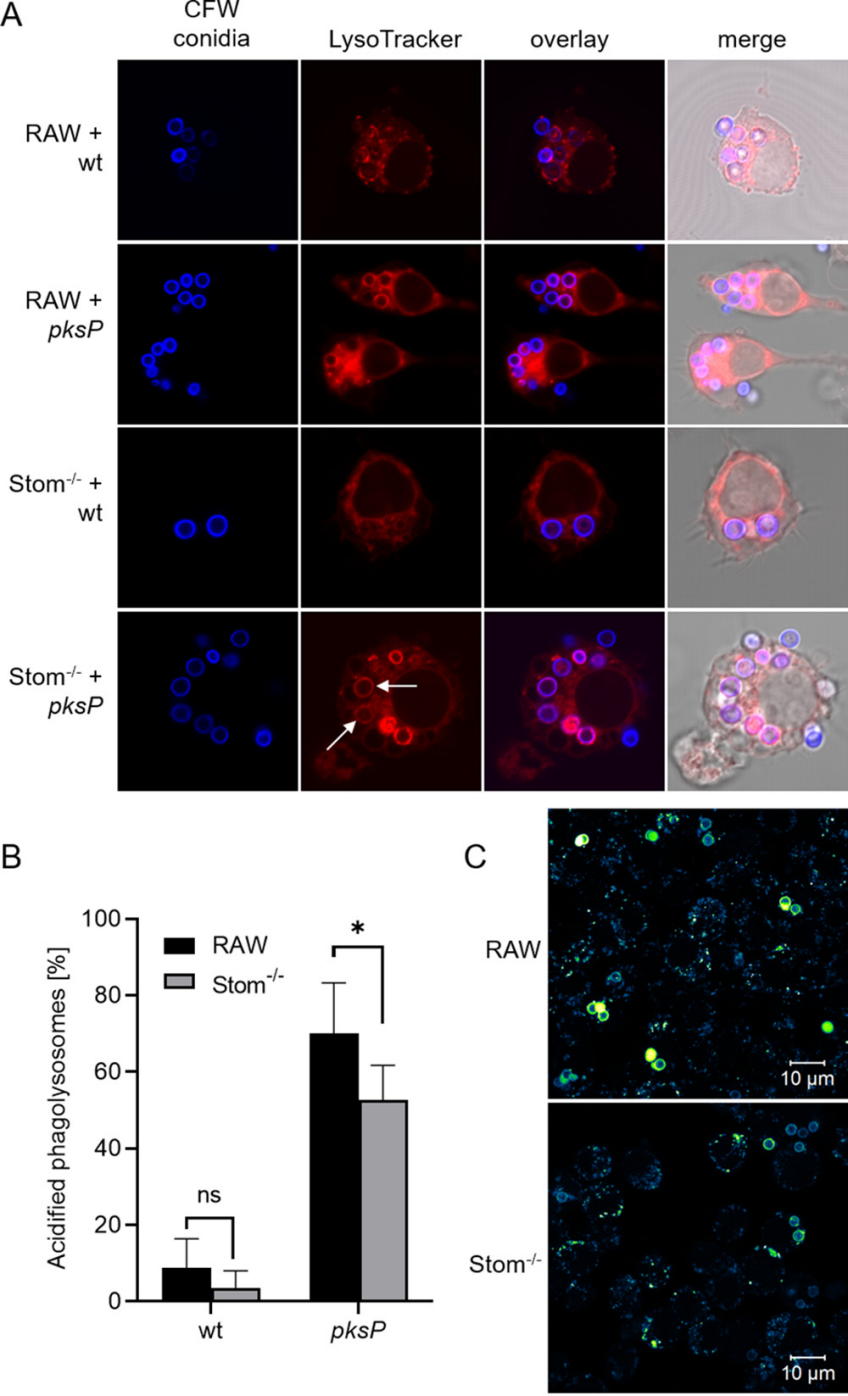
Impairment of phagosomal acidification by stomatin deficiency. (A) Detection of acidified phagosomes of RAW264.7 macrophages containing wild-type or *pksP* conidia using LysoTracker Red. Conidia were stained with CFW (blue). (B) Quantification of results in panel A. (C) Staining of phagosomes containing *pksP* conidia in Stom^−/−^ cells with LysoSensor.

vATPase-dependent acidification of phagosomes can be detected by using LysoTracker red (13), which shows red fluorescence of phagolysosomes. Here, a difference in acidification of phagosomes between wild-type and Stom^−/−^ macrophages was observed (Fig. 7A and B). As previously reported (13), virtually no fluorescence of phagosomes containing wild-type conidia was observed, in contrast to clear fluorescence seen in phago(lyso)somes containing *pksP* conidia. For stomatin-deficient macrophages, fluorescence of some, but not all, *pksP* conidia-containing phagosomes was reduced (Fig. 7A, arrows). To verify these data, we employed an additional sensor dye, i.e., LysoSensor yellow/blue, which allows visualization of gradual changes in pH in phagosomes. Depending on the pH, its emission wavelength shifts from blue (pH neutral) to yellow (acidic). RAW264.7 macrophages infected with *pksP* conidia showed bright yellow rings at the phagosome, indicating full acidification (Fig. 7C). In contrast, in Stom^−/−^ cells only some phagosomes stained yellow, but others remained blue or displayed a dotted ring-like structure, indicating impaired acidification. The acidification kinetics were found to be time dependent, similar to the phagocytosis rate (Fig. S7A). Stom^−/−^ macrophages reach amounts of acidified phagolysosomes similar to those of RAW264.7 cells only after 4 h of coincubation with *pksP* conidia. We then monitored whether this stomatin-dependent delay affects killing of phagocytosed conidia. However, no significant differences in overall survival of wild-type and *pksP* conidia were apparent when conidia were confronted with RAW264.7 and Stom^−/−^ macrophages for 6 h (Fig. S7B). This finding is in concordance with the result that the absence of flotillins, which significantly delays but not completely inhibits phagosomal acidification, did also not affect overall intracellular killing of conidia by macrophages (17). Finally, to analyze whether delayed intracellular processing of conidia impairs the immune response, secretion of the early proinflammatory cytokine tumor necrosis factor alpha (TNF-α) was determined (Fig. S7C). In contrast to infected RAW264.7 cells, Stom^−/−^ macrophages displayed reduced TNF-α levels after 24 h of coincubation with wild-type or *pksP* conidia. Stimulation with curdlan, a hydrophobic linear β-glucan, resulted in a strong cytokine secretion of both wild-type and Stom^−/−^ cells.

10.1128/msphere.00523-22.7 FIG S7 Impact of stomatin on acidification kinetics, fungal killing, and immune response. (A) Acidification kinetics of RAW264.7 wild-type and Stom^−/−^ macrophages was monitored after infection with wild-type and *pksP* conidia for 60 to 240 min. (B) Survival of wild-type and *pksP* conidia after phagocytosis by RAW264.7 and Stom^−/−^ macrophages. (C) Secretion of TNF-α by infected RAW264.7 and Stom^−/−^ macrophages was determined after 24 h. In addition, both wild-type and Stom^−/−^ cells were stimulated with curdlan. Download FIG S7, PDF file, 0.1 MB. Copyright © 2023 Goldmann et al. 2023 Goldmann et al. https://creativecommons.org/licenses/by/4.0/ This content is distributed under the terms of the Creative Commons Attribution 4.0 International license.

### Stomatin contributes to maturation of phagosomes to phagolysosomes.

**FIG 8:**
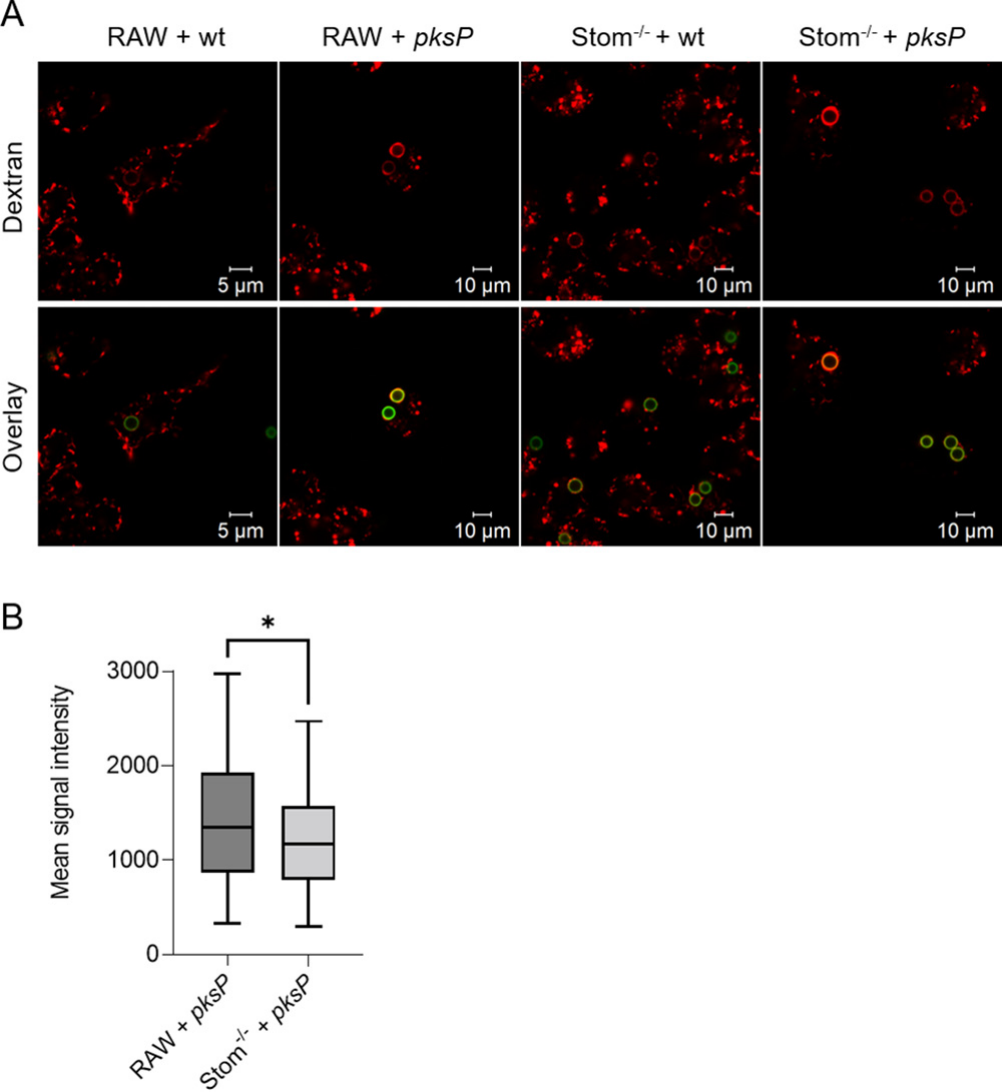
Monitoring of phagosomal maturation. (A) Macrophages infected with FITC-labeled conidia (green). The tetrarhodamine dextran stain (red) of the phagosome indicates fusion with lysosomes and maturation to phagolysosomes. (B) Comparison of mean fluorescence intensities of *pksP* conidia-containing phagolysosomes in RAW264.7 or Stom^−/−^ cells.

Due to the impaired dectin-1 recruitment to the phagosome, reduced vATPase assembly at the phagosomal membrane, and affected acidification, we hypothesized an involvement of stomatin in the maturation process of phagosomes. However, acidification of phagosomes in stomatin knockout cells was less impaired than that seen in a knockout of flotillins (17). Therefore, we monitored additional phagosomal maturation parameters such as the fusion of lysosomes with phagosomes. Such an analysis is feasible by loading lysosomes with tetramethylrhodamine dextran. A successful fusion of lysosomes with phagosomes is indicated by a rhodamine signal appearing in the produced phagolysosome. As shown in Fig. 8A and B, a significantly reduced rhodamine signal in phagosomes was observed for Stom^−/−^ macrophages compared to wild-type RAW264.7 cells after infection with *pksP* mutant conidia. As expected, because of the disruptive effect of DHN-melanin on the formation of lipid raft microdomains and thus maturation of phagosomes, infection with melanized wild-type conidia resulted in a low rhodamine signal in phagosomes in both RAW264.7 wild-type cells and Stom^−/−^ macrophages.

## DISCUSSION

Lipid rafts are small, highly dynamic microdomains in membranes that recruit and concentrate molecules involved in cellular signaling. In accordance with this function, membrane microdomains are also required for antifungal immunity (17, 41). A prominent and ubiquitously found protein in lipid raft microdomains is stomatin, which was suggested to be involved in the reorganization of the cytoplasmic membrane structure (19, 23, 24). Stomatin is known to interact with various ion channels and transporters, thereby modulating their activities (17, 23, 29, 30, 42, 43). Our data fully agreed with the localization of stomatin in various cellular membranes. Immunofluorescence analyses revealed accumulation of stomatin on both the cytoplasmic and phagolysosomal membranes of primary macrophages derived from bone marrow and macrophages of the RAW264.7 line. These data are also in line with proteomic studies of isolated phagolysosomes from RAW264.7 macrophages identifying stomatin (44).

We also discovered that stomatin is important for the initial phagocytosis of A. fumigatus conidia. This conclusion was drawn from experiments based on the successful inactivation of the *Stom* gene in the macrophage line RAW264.7. In Stom^−/−^ macrophages, reduced phagocytosis at initial phases was detected for pigmentless *pksP* mutant conidia and swollen wild-type conidia. For resting wild-type conidia, there was no difference in phagocytosis between Stom^−/−^ and wild-type cells. This observation well agrees with previous results showing that DHN-melanin of resting wild-type conidia covers immunogenic structures that are therefore less accessible to receptors (8) and that DHN-melanin inhibits formation of lipid rafts (17). Thus, an effect of the lack of the lipid raft microdomain component stomatin in association with melanized wild-type conidia, except that they were swollen and thus opened the melanin structure, was not expected.

An important receptor recognizing conidia is the C-type lectin receptor dectin-1, which binds the fungal surface polysaccharide β-1,3-glucan, which is more accessible on pigmentless *pksP* conidia than on melanized wild-type conidia (34, 45, 46). The presence of dectin-1 increases phagocytosis of *pksP* conidia and is associated with the production of cytokines and chemokines that trigger an inflammatory response (47). Dectin-1 was also shown to be a regulator of phagosomal maturation (37). Our data further support this finding, since we showed that stomatin also contributes to the enrichment of dectin-1 on the phagosomal membrane after phagocytosis of A. fumigatus conidia. Taken together, these data strongly suggest a regulatory connection of the PRR dectin-1 and the formation of flotillin-dependent lipid raft microdomains. Furthermore, as shown here, stomatin is required for full acidification of phagolysosomes and normal TNF-α levels produced by wild-type macrophages. Acidification of the phagolysosomal lumen depends on the vATPase proton pump (48). A direct link between lipid raft microdomains and assembly of the vATPase on the phagosomal membrane had been already reported (17, 40, 49). The vATPase complex was shown to colocalize with the lipid raft marker protein flotillin-1 (17, 50). Therefore, it seemed likely that stomatin is also required for the assembly and/or enrichment of the vATPase complex in maturing phagosomes. This assumption was confirmed by our immunofluorescence studies revealing that stomatin is involved in the recruitment of the cytoplasmic V_1_ subunit to the membranous V_0_ subunit to form a functional vATPase complex that is able to pump protons into the phagosomal lumen. We speculated whether the assembly of the vATPase is disturbed in the absence of stomatin or rather the fusion of phagosomes and lysosomes. Combined with the impaired localization of dectin-1 to the cytoplasmic membrane, our data suggest a role for stomatin in the fusion of lysosomes and likely other transport vesicles. In this case, stomatin-containing lipid raft microdomains act as fusion platforms.

The finding that the lack of stomatin only initially reduced phagolysosomal acidification also explains why overall intracellular killing of conidia in Stom^−/−^ macrophages was not affected after 6 h. The same observation for the overall intracellular killing of conidia was made in Flot^−/−^ BMDMs after 6 h (17).

Previously, we reported that conidial DHN-melanin interferes with formation of flotillin-dependent lipid raft microdomains in the phagolysosomal membrane (17). Surprisingly, the localization of stomatin on the membrane was not affected by DHN-melanin; also, the lack of stomatin apparently did not lead to major changes in lipid raft microdomains because staining for GM1 in Stom^−/−^ cells did not differ from that seen in wild-type cells. Therefore, this raises the question of the link between stomatin and flotillins. Both lipid raft markers belong to the SPFH domain proteins (51) and share several characteristics, such as a related topology and regulatory roles for signaling pathways (52–55). Like stomatin, flotillins are ubiquitously expressed and are conserved proteins involved in various cellular processes, such as membrane trafficking, phagocytosis, phagosomal maturation, and T-cell activation (56). Our data suggest that stomatin does not function as a chaperone like flotillins and caveolin, which apparently structure lipid raft microdomains, but rather contributes to positioning of certain proteins, such as dectin-1, in lipid rafts. In line with our hypothesis, in lipid raft microdomains we observed colocalization of stomatin and flotillin at the phagolysosomal membrane of RAW264.7 macrophages after phagocytosis of A. fumigatus conidia (Fig. S8).

10.1128/msphere.00523-22.8 FIG S8 Immunofluorescence to monitor colocalization of flotillin-1 and stomatin on isolated phagolysosomes of macrophages after infection with *pksP* conidia. Download FIG S8, PDF file, 0.1 MB. Copyright © 2023 Goldmann et al. 2023 Goldmann et al. https://creativecommons.org/licenses/by/4.0/ This content is distributed under the terms of the Creative Commons Attribution 4.0 International license.

Collectively, our data are in accordance with the following model. In stomatin knockout cells, lipid raft microdomains are still present. Stomatin is likely important for positioning of proteins in lipid rafts and plays a role in fusion processes during membrane recycling and maturation of phagolysosomes. This is achieved by a stomatin-dependent formation of larger lipid raft microdomains that, in addition, allow for more efficient fusion of phagosomes with lysosomes and thus for an increase of both functional vATPase complexes and localization of dectin-1 proteins on mature phagolysosomes. In line with this, accumulation of dectin-1 on phagosomal membranes was shown to be dependent on acidification of phagosomes (37), and dectin-1 colocalized to lipid raft microdomains (57). Stomatin may also be involved in the direct incorporation of proteins into lipid raft microdomains, triggered in part by the fusion of phagosomes with lysosomes. In the future, further studies, e.g., involving infection of stomatin-deficient mice, would be highly interesting to prove the *in vivo* importance of stomatin in host-pathogen interactions.

### Conclusion.

The data presented here provide new insights into the important role of the integral membrane protein stomatin in the immune response against human-pathogenic fungi. We provide evidence that stomatin plays a crucial role in the quantitative localization of dectin-1 and vATPase in cytoplasmic and/or phagosomal membranes, thereby impacting phagocytosis and intracellular processing of A. fumigatus conidia.

## MATERIALS AND METHODS

### Cell lines.

The murine macrophage line RAW264.7 (ATCC TIB-71) was cultivated in Dulbecco’s modified Eagle medium (DMEM; Gibco) supplemented with 10% (vol/vol) fetal bovine serum (FBS; GE Healthcare Life Sciences), 1% (wt/vol) UltraGlutamine (Gibco), and 27.5 μg/mL gentamicin sulfate (Gibco) at 37°C and 5% (vol/vol) CO_2_ in a humidified chamber. When required, the medium was further supplemented with puromycin (7 μg/mL for selection of clones or 6 μg/mL for maintenance of cells; InvivoGen) and/or hygromycin (100 μg/mL for selection of clones or 70 μg/mL for maintenance of clones; InvivoGen). The human kidney epithelial cell line Lenti-X 293T (TaKaRa) was maintained in DMEM supplemented with 10% (vol/vol) FBS, 1% (wt/vol) UltraGlutamine, 1% (vol/vol) sodium bicarbonate (Gibco), and 1% (vol/vol) penicillin-streptomycin (Gibco).

### Cultivation, staining, and fixation of A. fumigatus.

Wild-type A. fumigatus strain ATCC 46645 and the corresponding pigmentless *pksP* mutant (14) were cultivated on Aspergillus minimal medium (AMM) agar plates as described elsewhere (58). Conidia were grown for 5 days at 37°C and harvested with 0.9% (wt/vol) NaCl–0.01% (vol/vol) Tween 20. Conidia were labeled with calcofluor white (CFW; Sigma-Aldrich) or fluorescein isothiocyanate (FITC; Sigma-Aldrich) as previously described (13). To achieve swollen conidia, 4 × 10^8^ A. fumigatus wild-type conidia were inoculated in 10 mL RPMI 1640 for 5 h at 200 rpm and 37°C. Subsequently, the conidial suspension was centrifuged at 1,000 × *g* for 5 min. The supernatant was discarded and the swollen conidia were resuspended in 3.7% formaldehyde in phosphate-buffered saline (PBS) and fixed for 30 min at 4°C. After fixation, the conidia were washed three times by centrifugation at 1,000 × *g* for 5 min and resuspension in PBS.

### Infection experiments.

For infection experiments, macrophages were seeded in 24-well plates with coverslips at a density of 3× 10^5^ cells/well and inspected microscopically. For further analysis of isolated phagosomes, 4 × 10^6^ cells/well were seeded in 4-well plates. The cells were infected with conidia at a multiplicity of infection (MOI) of 2 or 5, as indicated. Synchronization of infection was achieved by incubating cells at 4°C for 30 min or by centrifugation for 5 min at 200 × *g*. Infection was allowed to proceed for 30 to 240 min at 37°C in a humidified chamber at 5% (vol/vol) CO_2_. The samples were then processed according to the requirements of the respective assay.

### Generation of a RAW264.7 stomatin knockout cell line.

To perform gene editing in RAW264.7 macrophages, the Lenti-X CRISPR/Cas9 system (TaKaRa) was used. First, RAW264.7 cells with stable production of Cas9 were generated according to the manufacturer’s protocol. In brief, Lenti-X 293T cells were transfected with vector pLVX-puro-Cas9 to produce Cas9 lentiviral particles. Lentiviral titer was measured by the Lenti-X p24 rapid titer kit (TaKaRa). Then, RAW264.7 macrophages were transduced with Cas9-lentivirus particles at an MOI of 5 and monoclonal cells were obtained by selection with puromycin (7 μg/mL). To target the stomatin (*Stom*) gene, single guide RNAs (sgRNAs) were designed with the online tool ChopChop (https://chopchop.cbu.uib.no/). Oligonucleotides CACCGAGAGTCATCATCTTTAGAC and AAACGTCTAAAGATGATGACTCTC, corresponding to the target sgRNA, were annealed and cloned into vector pLVX-hyg-sgRNA. Lenti-X 293T cells were transfected with the resulting vector pLVX-hyg-Stom-sgRNA. As a result, they produced lentivirus particles encoding *Stom* sgRNAs. Then, RAW264.7 macrophages with stable production of Cas9 were transduced with isolated *Stom* sgRNA-lentivirus particles. Cell clones were obtained by selection of cells with hygromycin (100 μg/μL). After several passages, stable clones were screened for gene editing events resulting in the lack of stomatin production. A knockout of stomatin was verified by Western blotting. At the DNA level, deletion of a single nucleotide in the stomatin gene was verified by DNA sequence analysis. For this purpose, a DNA fragment comprising the sgRNA target region was amplified by PCR using primers AATAGAGCAAACAACAGGAGGC and GAGTACTGACCTGGTCCTTTGG and the Phusion Flash high-fidelity PCR master mix (Thermo Scientific).

### Isolation of murine BMDMs.

C57BL/6J mice were supplied by Charles River (Sulzfeld, Germany). Dectin-1^−/−^ mice were kindly provided by Ilse Jacobsen, Jena, Germany. Bone marrow cells from C57BL/6J mice and dectin-1^−/−^ mice were isolated from femurs and tibias of 12- to 16-week-old mice as described elsewhere (59). Isolated bone marrow was treated with ACK lysis buffer (Gibco) to lyse red blood cells. Bone marrow-derived macrophages (BMDMs) were differentiated as described previously (17) by cultivation of bone marrow cells for at least 5 days in DMEM supplemented with 20 ng/mL macrophage colony-stimulating factor (M-CSF; Peprotech), 10% (vol/vol) FBS, 1% (wt/vol) UltraGlutamine, and 0.05 M 2-mercaptoethanol (Gibco).

### Cell lysate preparation and isolation of Aspergillus-containing phagosomes.

To obtain protein extracts, cells were lysed with radioimmunoprecipitation assay (RIPA) buffer (Sigma-Aldrich). For this purpose, 1 × 10^6^ cells were sedimented by centrifugation, resuspended in 100 μL RIPA buffer, and centrifuged for 15 min at 16,000 × *g* and 4°C. The supernatant contains cellular proteins.

The isolation of conidia-containing phagosomes from RAW264.7 macrophages or primary macrophages was performed as described previously (60). In brief, after infection with conidia, cells were harvested with PBS and centrifuged at 200 × *g* for 5 min at 4°C. The cell pellet was resuspended in 1 mL homogenization buffer (250 mM sucrose, 3 mM imidazole [pH 7.4]) containing protease inhibitor (c*O*mplete; Roche). Cell lysis was carried out on ice by passing the cell suspension 20 times through a 26-gauge needle. To release the rigor mortis actin-myosin interaction, the homogenates were incubated with 10 mM ATP for 15 min with rotation at 4°C. Then, the homogenate containing phagosomes was layered over 500 μL Biocoll (Merck) and centrifuged at 600 × *g* for 20 min. The pellet, consisting of the conidia-containing phagosomes, was washed twice with PBS and resuspended in PBS for further analyses.

### SDS-PAGE and Western blot analysis.

Protein concentrations were determined by Bradford assay as described previously (61). Protein extracts of equal amounts were separated by SDS-PAGE (NuPAGE 4 to 12% [wt/vol] bis-Tris protein gels; Invitrogen) and transferred to a polyvinylidene difluoride (PVDF) membrane (iBlot2 PVDF ministacks; Invitrogen). The membrane was blocked with 5% (wt/vol) milk powder at room temperature for 1 h and incubated overnight at 4°C with the primary antibody. As primary antibodies, mouse anti-Cas9 antibody (MA1-201; Invitrogen), rabbit anti-stomatin (ab166623; Abcam), and rabbit anti-vimentin as a loading control (5741; Cell Signaling Technology) were used with a dilution of 1:500 (Cas9) or 1:2,000 (stomatin and vimentin). After incubation with horseradish peroxidase (HRP)-conjugated IgG antibodies, goat anti-rabbit IgG-HRP (ab6721; Abcam) and anti-mouse IgG-HRP (7076; Cell Signaling Technology), both diluted 1:2,000, were added for 1 h at room temperature. Detection was performed using the 1-Step Ultra TMB-Blotting solution (Thermo Scientific).

### Quantitation of the phagocytosis ratio, acidification of phagosomes, and recruitment of lipid raft microdomains.

To analyze the phagocytosis ratio, macrophages were infected with FITC-labeled wild-type or *pksP* conidia of A. fumigatus at an MOI of 5. To synchronize phagocytosis, samples were centrifuged for 5 min at 200 × *g*. To monitor the kinetics of phagocytosis, coincubation was started in a CO_2_ incubator at 37°C and the samples were incubated for 30 min, 60 min, and 120 min. The process was stopped by washing with 1 mL ice-cold PBS. Nonphagocytosed conidia were not removed during the washing steps since after centrifugation and incubation they adhered to macrophages and/or the plate. Nonphagocytosed, extracellular conidia were counterstained with 0.25 mg/mL CFW for 15 min. Cells were washed twice with PBS and afterwards fixed for 15 min with 3.7% (vol/vol) formaldehyde-PBS. Cells were stained for 20 min with CellMask red (Life Technologies) at 37°C. Following a final washing step, coverslips were transferred to glass slides and imaged using an LSM 780 confocal microscope (Zeiss). In each biological replicate, about 200 conidia were counted and the quantity of phagocytosed conidia was compared to the number of nonphagocytosed conidia. Comparison of phagocytosed wild-type and *pksP* conidia was performed by maintaining the same settings for the applied fluorescence channels during the image processing workflow. This included the choice of fluorescence threshold values, as well as the scale of smoothing for both channels (17).

For measuring acidification of phagosomes prior to infection with conidia, macrophages were prestained with 50 nM LysoTracker red DND-99 (Life Technologies) for 1 h. Then, the medium was exchanged with fresh medium with LysoTracker red DND-99. Macrophages were infected with CFW-labeled wild-type or *pksP* conidia at an MOI of 2, and phagocytosis was synchronized by centrifugation. In a period of 1 to 4 h of coincubation, cells were fixed with 3.7% (vol/vol) formaldehyde-PBS as described above and imaged using an LSM 780 microscope. For the evaluation of the phagosomal acidification, 100 conidia-containing phagosomes were analyzed; a strong red signal around the conidia indicated a decrease in pH. For further characterization of the phagosomal acidification, a second dye, LysoSensor yellow/blue DND-160 (Life Technologies), was applied. Macrophages were preloaded with 1 μM LysoSensor yellow/blue DND-160 for 30 min. Afterwards, the medium was exchanged with fresh medium with LysoSensor yellow/blue DND-160 and cells were infected with conidia (MOI of 2) and coincubated for 2 h, as described above. After fixation, the samples were microscopically analyzed for acidification of the phagosomes. This was indicated by a shift of the emission spectrum from blue (neutral pH) to yellow (acidic pH).

To visualize GM1 (monosialotetrahexosylganglioside) in phagosomal membranes, macrophages were incubated with 1.5 μM Alexa Fluor 647-conjugated cholera toxin B (CTB; Life Technologies) for 1 h and afterwards infected with FITC-labeled A. fumigatus conidia at an MOI of 2. Phagocytosis was allowed to proceed for 2 h after synchronization by centrifugation. For microscopic analysis, cells were fixed with 3.7% (vol/vol) formaldehyde-PBS as described above. One hundred conidia-containing phagosomes were evaluated for GM1 recruitment, visualized by an accumulation of the CTB-Alexa Fluor 647 dye in the phagosomal membrane. All values represent means ± standard deviations (SD) of three biological replicates.

### Immunofluorescence and microscopy.

Cells were fixed with 3.7% (vol/vol) formaldehyde-PBS for 15 min or, if indicated, with methanol-acetone (80% [vol/vol] to 20% [vol/vol]) for 20 min. Cells were then permeabilized with 0.1% (vol/vol) Triton X-100–PBS for 10 min. Isolated phagosomes were not permeabilized. Macrophages and isolated phagosomes were blocked with 5% (vol/vol) normal goat serum (Thermo Fisher Scientific), 2% (wt/vol) bovine serum albumin (BSA), and 0.3 M glycine-PBS for 30 min before overnight incubation at 4°C with the primary antibody in 2% (wt/vol) BSA-PBS. As primary antibodies, rabbit anti-stomatin (ab166623; Abcam), rabbit anti-ATP6V1B2 (ab73404; Abcam), goat anti-dectin-1/CLEC7 (AF1756; R&D Systems), and rabbit anti-dectin-1 (PA5-34382; Invitrogen) were used at a concentration of 1:500 (stomatin and vATPase) or 1:100 (dectin-1). The incubation with secondary antibodies, goat anti-rabbit IgG DyLight 633 (35562; Thermo Fisher Scientific), goat anti-rabbit IgG Alexa Fluor 532 (A11009; Life Technologies), and donkey anti-goat Alexa Fluor 488 (A11055; Life Technologies), was carried out at room temperature for 1 h according to the manufacturer’s specifications. Samples were finally visualized using an LSM 780 confocal microscope with Airyscan technology and ZEN software (Zeiss).

### Measurement of phagosomal maturation.

To monitor phagosomal maturation, macrophages were seeded at a density of 3 × 10^5^ cells/well on coverslips and stained overnight with 60 μg/mL tetramethylrhodamine dextran (molecular weight [MW], 3,000; Invitrogen). On the next day, the medium was exchanged to standard cultivation medium and cells were infected with FITC-labeled A. fumigatus conidia at an MOI of 2. After synchronization of phagocytosis by centrifugation and incubation for 2 h at 37°C in a CO_2_ incubator, fusion of phagosomes and lysosomes was evaluated by microscopy. Fusion was detected by an increased accumulation of tetramethylrhodamine dye in conidia-containing phagosomes. For quantification, the mean fluorescence intensity of 100 phagosomes was calculated with the Zen software of the LSM 780 microscope.

### Killing assay.

To quantify the survival of A. fumigatus conidia after phagocytosis, RAW264.7 and Stom^−/−^ macrophages were seeded at a density of 1 × 10^6^ cells/well in a 6-well plate 1 day prior to infection. On the following day, cells were infected with wild-type or *pksP* conidia at an MOI of 2. Phagocytosis was synchronized by centrifugation for 5 min at 200 × *g*, and coincubation was allowed for 6 h at 37°C and 5% (vol/vol) CO_2_. The samples were harvested in Milli-Q water with 0.1% (vol/vol) Tween. Serial dilutions were plated on Sabouraud agar plates. CFU derived from conidia were counted after incubation for 24 h at 37°C and an additional incubation overnight at room temperature.

### Flow cytometry.

To analyze dectin-1 receptor expression on the surface of macrophages by flow cytometry, 10^6^ cells were pelleted in a 1.5-mL tube by centrifugation for 5 min at 300 × *g* and 4°C. The pellet was washed once with 1% (wt/vol) BSA in PBS plus 0.2 mM EDTA (FACS buffer) and subsequently resuspended in 100 μL FACS buffer containing 10% (vol/vol) FcR blocking reagent (Miltenyi Biotec) and 1% (vol/vol) Viobility 405/520 fixable dye (Miltenyi Biotec). The cell suspension was incubated for 10 min on ice, followed by addition of 0.5 μg either the anti-dectin-1 antibody (phycoerythrin [PE] anti-mouse CD369 [dectin-1/CLEC7A] antibody; BioLegend) or the respective isotype control (PE rat IgG1, κ isotype control antibody; BioLegend) and further incubation on ice for 30 min. The cells were washed 3 times by centrifugation (at 300 × *g* and 4°C for 5 min) and resuspension in 100 μL FACS buffer. After the last washing step, the cells were resuspended in 200 μL FACS buffer and measured by flow cytometry (BD LSR Fortessa; BD Biosciences). Data were analyzed by FlowJo software (BD Biosciences).

### Cytokine measurement.

Cells were seeded at a density of 3 × 10^5^/well in a 24-well plate and infected with wild-type or *pksP* conidia at an MOI of 5 or were treated with 100 μg/mL curdlan (β-1,3-glucan; Sigma-Aldrich). After synchronization of infection by centrifugation, the samples were incubated for 24 h at 37°C with 5% (vol/vol) CO_2_.The supernatants were collected and processed according to the enzyme-linked immunosorbent assay (ELISA) MAX deluxe set mouse TNF-α kit (BioLegend). Results were calculated by a 4-parameter sigmoidal curve fit.

### Image analysis.

Isolated phagosomes were imaged in transmitted light (TL) and fluorescence mode of confocal microscopy to reveal the localization of the phagosomes and their vATPase level. Images were analyzed with a custom-designed algorithm written in the graphical programming language JIPipe (https://www.jipipe.org/). The targeted measurements consider the intensity of the vATPase fluorescence signal detected within the phagosomes. Phagosomes were identified from the TL images, which provided a label-free way to reliably segment all phagosomes independently of their vATPase fluorescence signal. The fluorescence signal specific for vATPase was not suitable for such segmentation, because the production of the proteins of the complex varied according to the experimental conditions and thus could not be algorithmically identified. At the same time, the TL images provided a suitable platform for the segmentation process. On the other hand, TL images did not allow the segmentation of the isolated phagosomes based on pixel intensity, because the gray value of the image pixels within the phagosome areas varied according to laser intensity and relative light phase. Thus, we utilized Cellpose (62), a deep learning (DL)-based generalized image segmentation framework that was originally trained on a very large number of cells and cell-like objects. Our analysis workflow was written entirely in JIPipe, using specific nodes dedicated to training (see Fig. S1 to S4 in the supplemental material) and applying Cellpose tools directly within the workflow.

10.1128/msphere.00523-22.1 FIG S1 JIPipe workflow of applying the Cellpose model to segment phagosomes in transmitted light images and to measure the corresponding vATPase fluorescence in these regions of interest. The image properties (folders and subfolders, replicate and strain names, and the image file name) were identified and added to the image objects in the form of JIPipe annotations (black round-corner rectangle). The scale images step (black rhomboid) unified the pixel size in all images, a step made necessary by having confocal images of various spatial resolutions. The channel assignment step (black rhomboid) separates the transmitted light (gray rectangle) and fluorescence channels (red rectangles), the latter corresponding to the vATPase signal. The transmitted light images were then corrected for illumination inhomogeneities (grey rhomboid) and plugged into the Cellpose application nodes (green oblique rectangles). Here, one Cellpose node each was used to apply the default model and the transferred learning-based model, respectively. The latter received the trained model from the Cellpose training compartment (blue oval and rectangle) as shown in Fig. S2. The segmented phagolysosomes were filtered based on their size and shape (gray hexagons): only objects between 70 and 500 pixels and with circularity above 0.5 were considered for further analysis. Since each image had two segmentations (default and transfer learning model based), a manual decision step was inserted as a next step (gray diamond) to decide which model to use for the measurements. The selected model provided the ROIs (regions of interest, magenta wavy rectangle) that were used in the next step to measure the vATPase fluorescence intensity in the phagolysosomes (black oval). The intensity values within the ROIs projected onto the vATPase image (red rhomboid) were saved for further analysis, including intensity distribution histograms under various conditions. Download FIG S1, PDF file, 0.1 MB. Copyright © 2023 Goldmann et al. 2023 Goldmann et al. https://creativecommons.org/licenses/by/4.0/ This content is distributed under the terms of the Creative Commons Attribution 4.0 International license.

First, all images were tested with the default Cellpose model (Fig. S2). Cellpose can be applied either by using its default pretrained model or by training the underlying DL network either from scratch or via transfer learning. We combined the application of the default model and the transfer learning-based modified model. After examining the outcome, 1,031 isolated phagosomes were manually outlined from a subset of the images where the default model failed, in order to provide a training database for Cellpose transfer learning. The application of the latter technique was necessary because the default model sometimes failed either by missing the darker-toned phagosomes (Fig. S3A and B) or by producing false-positive predictions (Fig. S3C and D), whereas the custom-trained model performed very well with the problematic images (Fig. S3E and F).

10.1128/msphere.00523-22.2 FIG S2 Node array of the Cellpose preprocessing compartment. Download FIG S2, PDF file, 0.2 MB. Copyright © 2023 Goldmann et al. 2023 Goldmann et al. https://creativecommons.org/licenses/by/4.0/ This content is distributed under the terms of the Creative Commons Attribution 4.0 International license.

10.1128/msphere.00523-22.3 FIG S3 Illustrative images demonstrating the selection between the default and transfer learning-based trained Cellpose models. In the horizontal pairs on images, the left and right panels show the segmentation predicted by the default or transfer learning-based Cellpose models, respectively (yellow outlines). In panels A and B, the default model outperformed the trained model, whereas in panels C and D and panels E and F, transfer learning was necessary to recognize the phagolysosomes that appear in a darker phase in the transmitted light images. Download FIG S3, PDF file, 0.6 MB. Copyright © 2023 Goldmann et al. 2023 Goldmann et al. https://creativecommons.org/licenses/by/4.0/ This content is distributed under the terms of the Creative Commons Attribution 4.0 International license.

Finally, the default and the trained models were applied in parallel (Fig. S4) and the best-fitting model was selected for each image. The comparison of the two models indicated the success of this combined approach.

10.1128/msphere.00523-22.4 FIG S4 Node array of the Cellpose training compartment for Fig. S3. Download FIG S4, PDF file, 0.3 MB. Copyright © 2023 Goldmann et al. 2023 Goldmann et al. https://creativecommons.org/licenses/by/4.0/ This content is distributed under the terms of the Creative Commons Attribution 4.0 International license.

Data acquired earlier in the project were quantified using a project-specific version of the ImageJ-based analysis framework ACAQ (63). Intensity-based contrast in the TL images was created using a Hessian filter as described previously (63, 64), thus avoiding the need for fluorescence labeling of the membrane. The Hessian-filtered TL images were processed further to segment the individual vesicles and to measure the mean fluorescence intensity within the resulting regions of interest.

### Quantification and statistical analysis.

If not stated otherwise, at least 100 events per sample of three biological replicates were analyzed. Data are presented as means ± SD. *P* values were calculated by a two-tailed Student *t* test. In figures, statistical significance is indicated as follows: *, *P < *0.05; **, *P < *0.01; and ***, *P < *0.001.
