# Supplementary figures and images for: The Lipid Raft-Associated Protein Stomatin Is Required for Accumulation of Dectin-1 in the Phagosomal Membrane and for Full Activity of Macrophages against Aspergillus fumigatus
Source: mSphere. 2023 Jan 31;8(1):e00523-22. doi: 10.1128/msphere.00523-22 (PMC9942578; doi:10.1128/msphere.00523-22)

Supplementary Figure 5

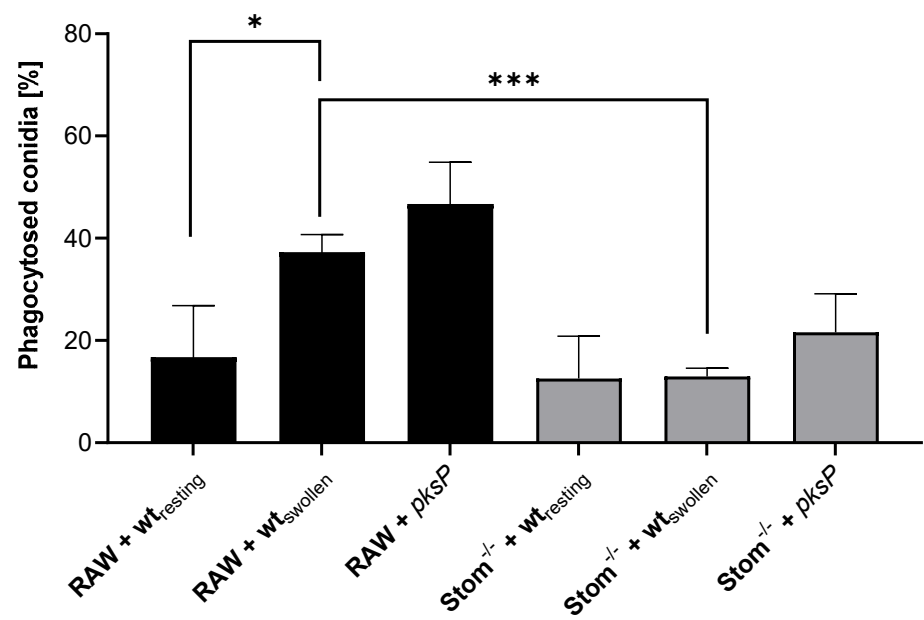

Supplement: FIG S5 [file msphere.00523-22-s0005.pdf]

Supplementary Figure 6

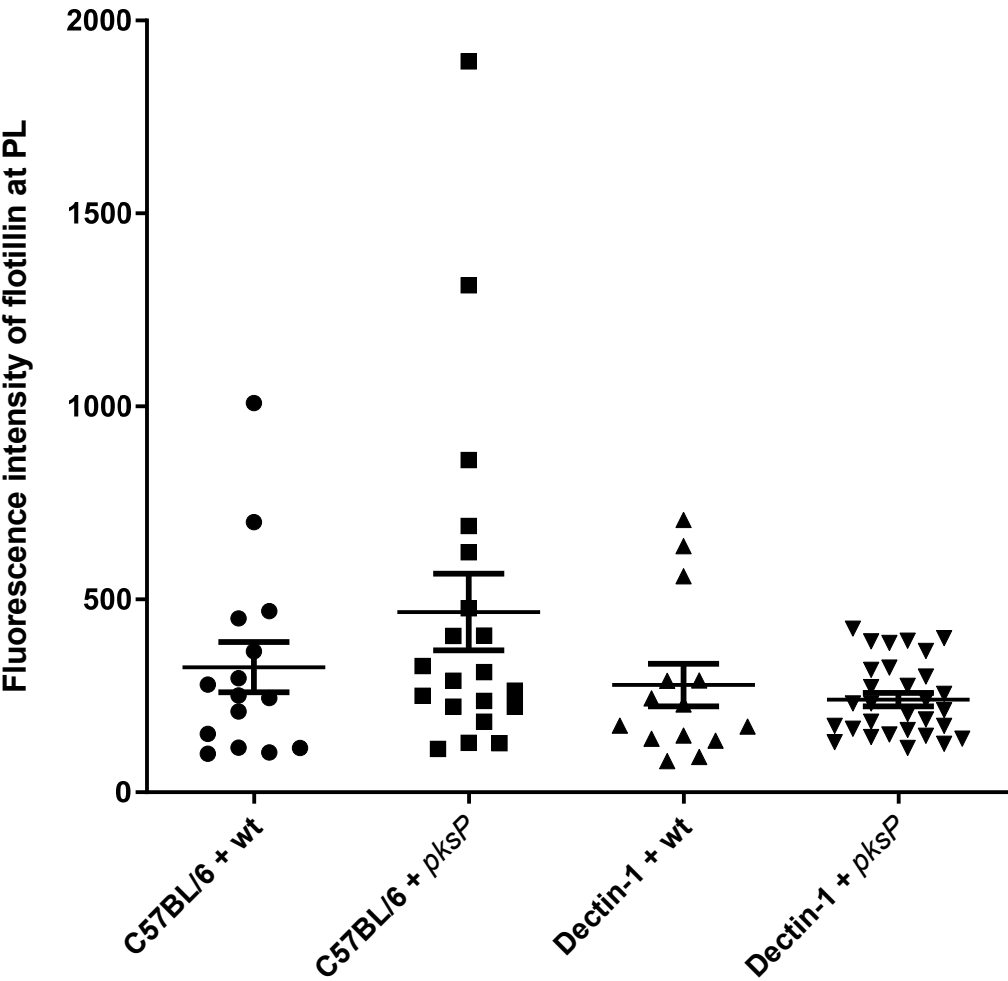

Supplement: FIG S6 [file msphere.00523-22-s0006.pdf]

# Supplementary Figure 7

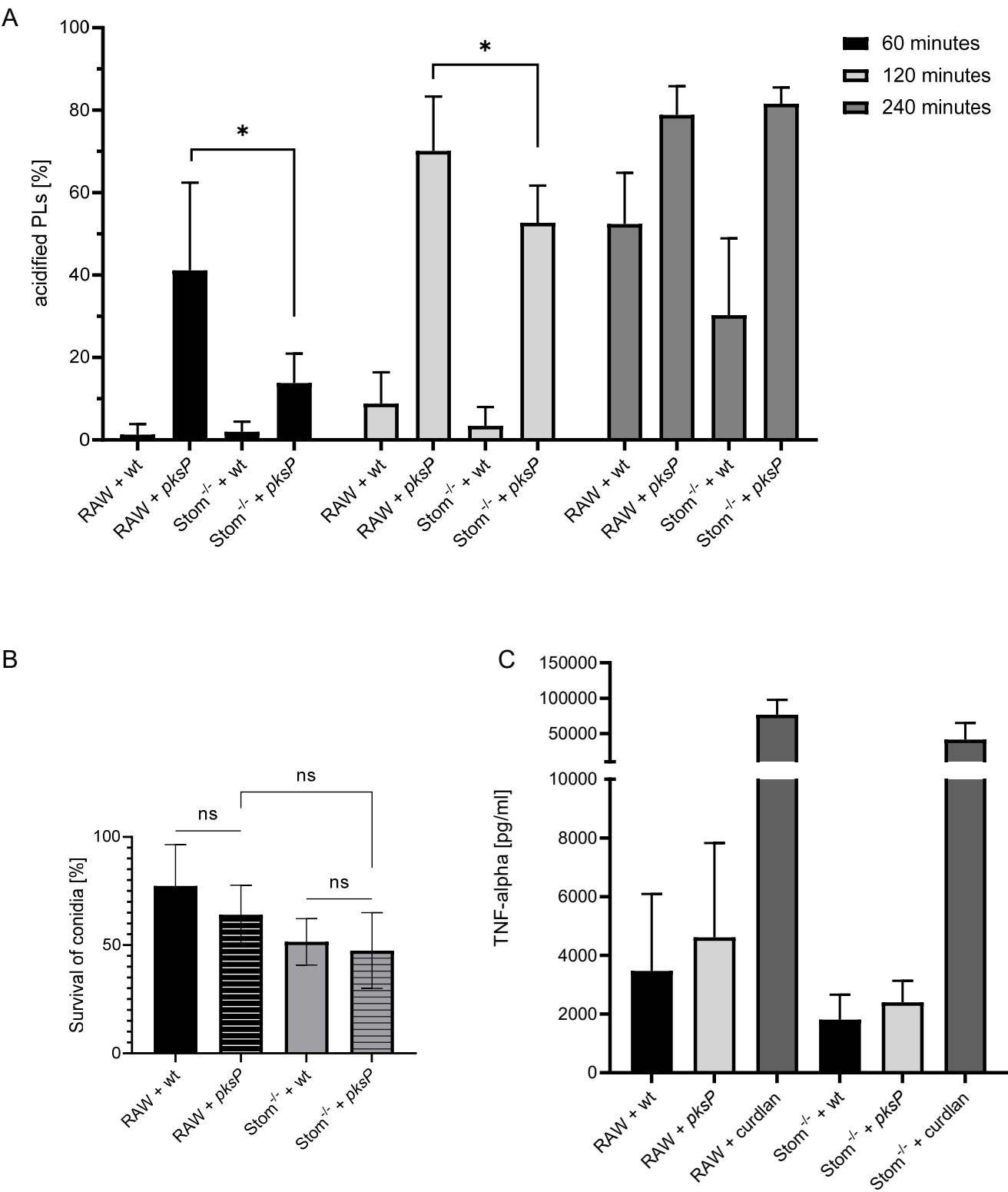

Supplement: FIG S7 [file msphere.00523-22-s0007.pdf]

Supplementary Figure 8

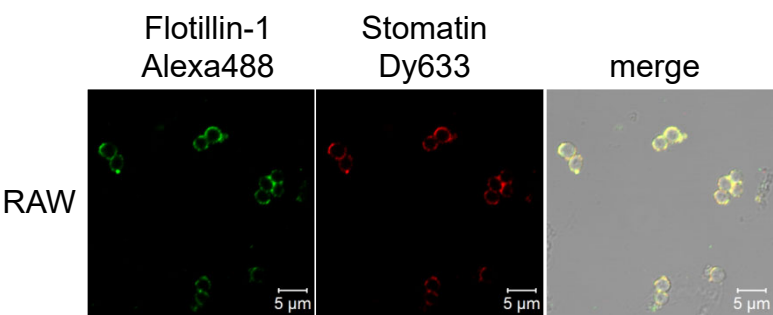

Supplement: FIG S8 [file msphere.00523-22-s0008.pdf]

## Supplementary Figure 1

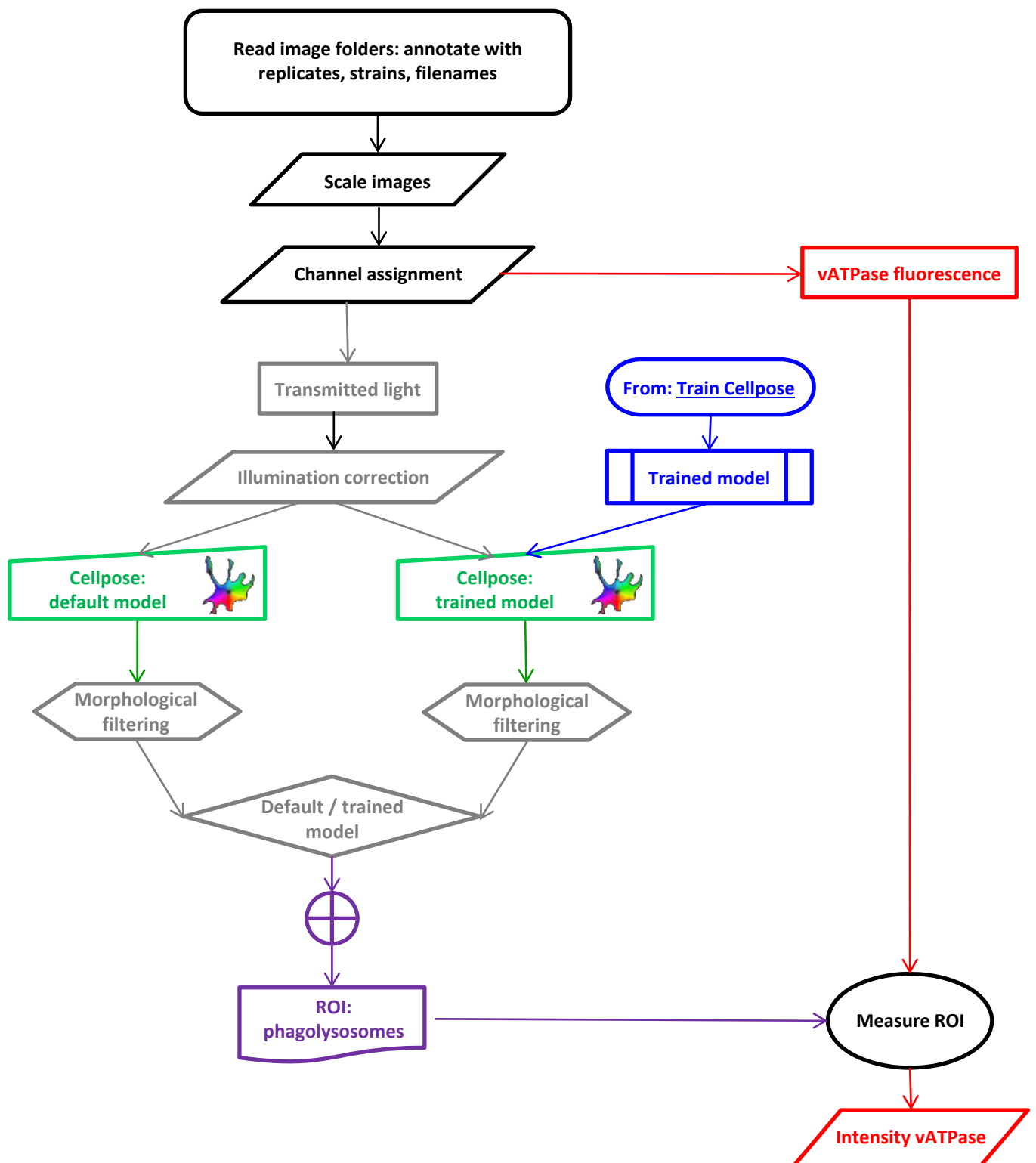

Supplement: FIG S1 [file msphere.00523-22-s0001.pdf]

Supplementary Figure 2

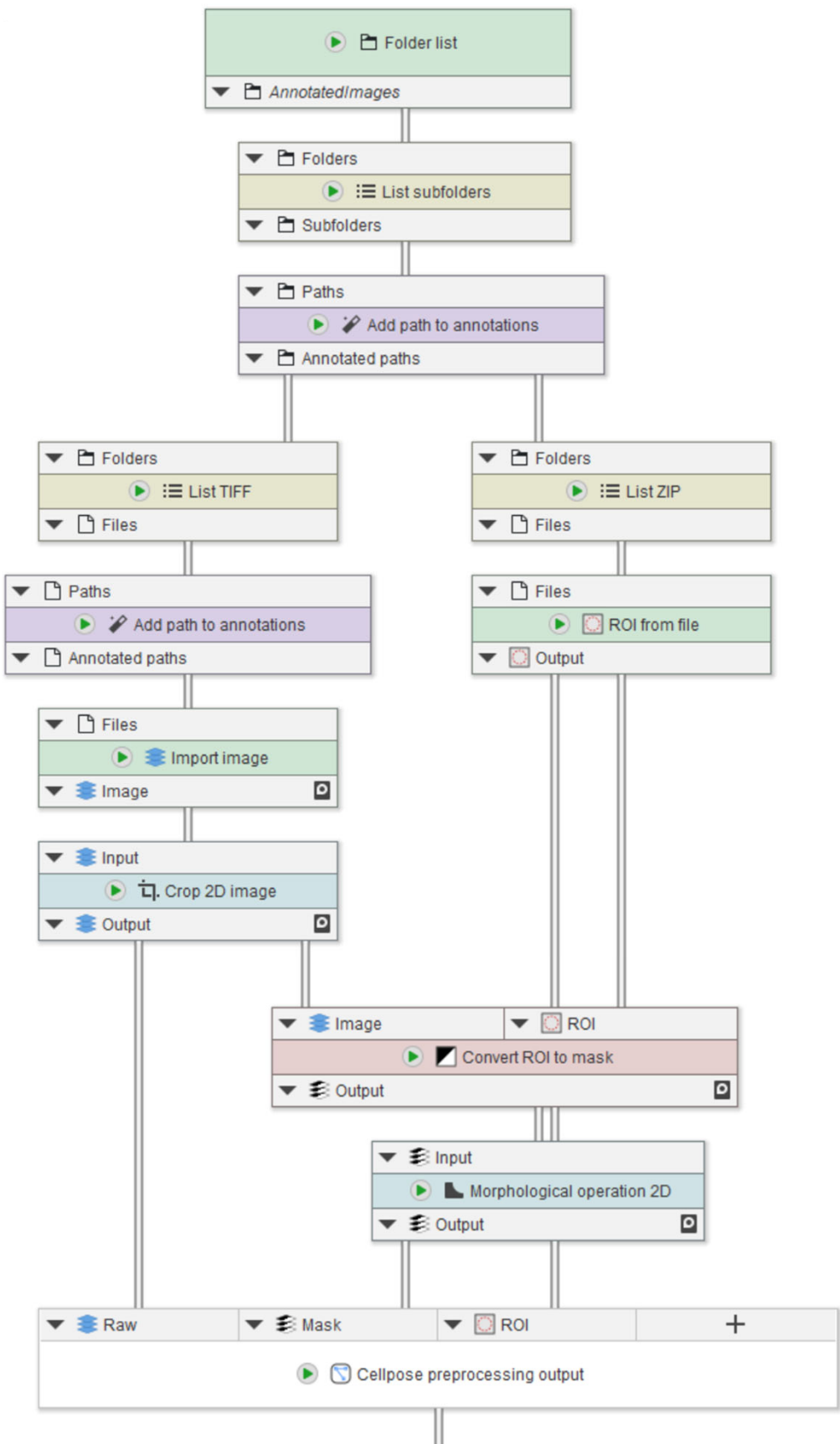

Supplement: FIG S2 [file msphere.00523-22-s0002.pdf]

Supplementary Figure 3

Default model

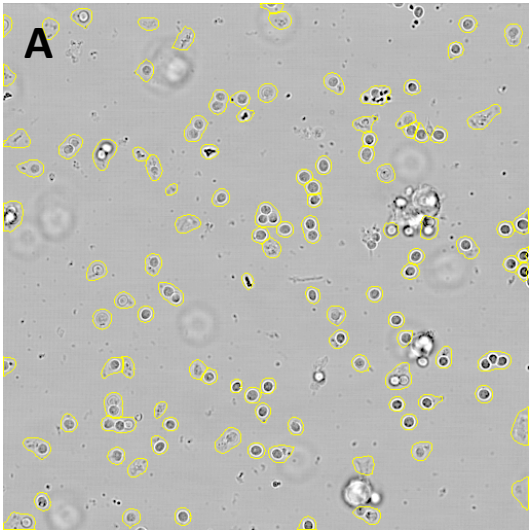

Transfer learning model

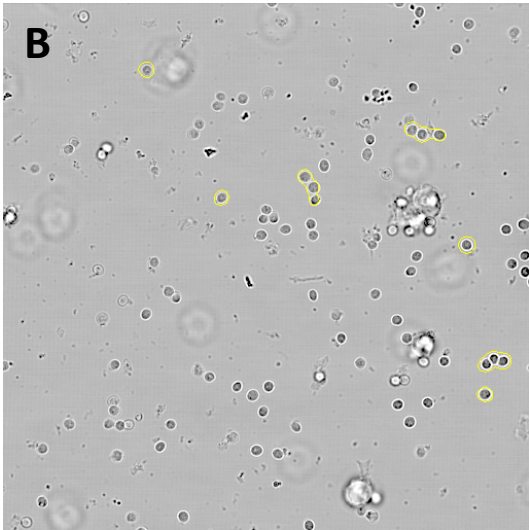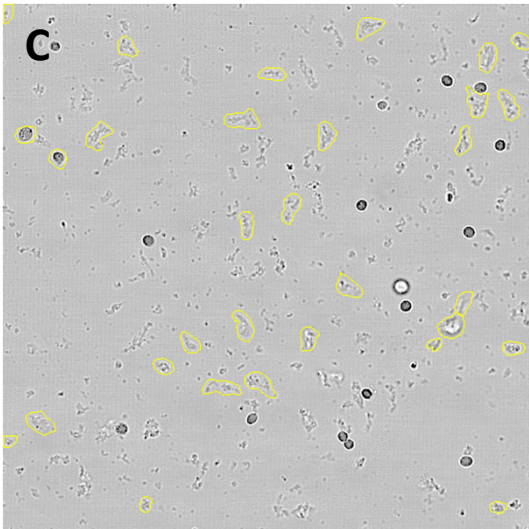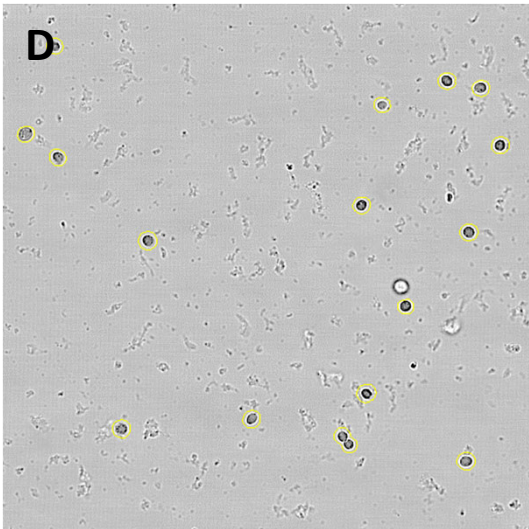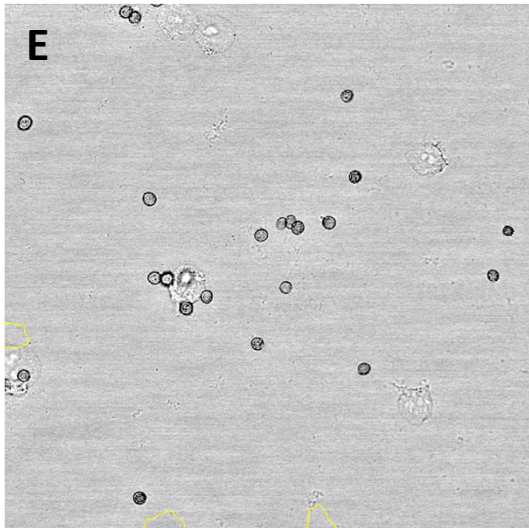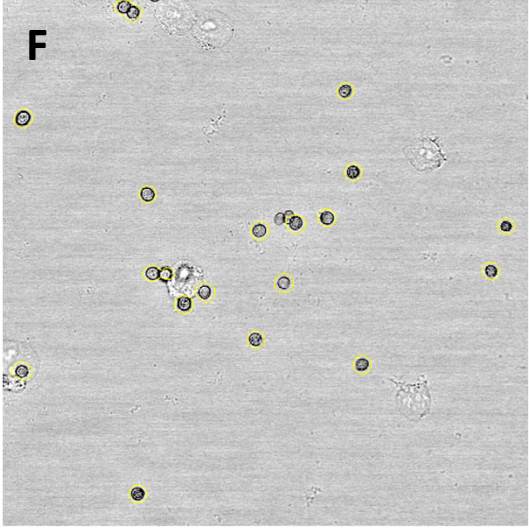

Supplement: FIG S3 [file msphere.00523-22-s0003.pdf]

Supplementary Figure 4

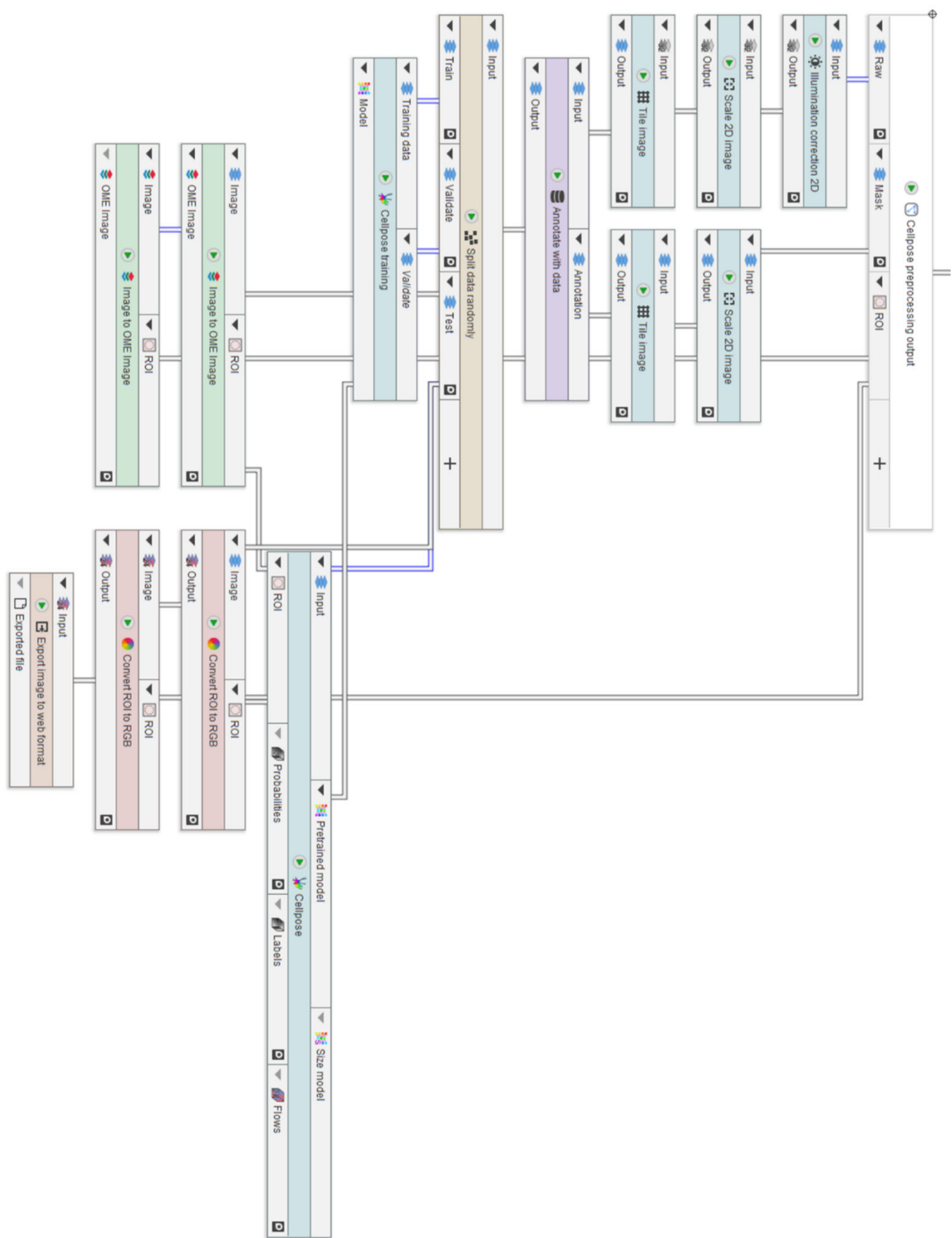

Supplement: FIG S4 [file msphere.00523-22-s0004.pdf]
